# Initial Experience With Robotic Mitral Valve Replacement: Results From a Single Centre

**DOI:** 10.1093/icvts/ivag002

**Published:** 2026-01-08

**Authors:** Ersin Kadiroğulları, Zihni Mert Duman, Salih Güler, Zinar Apaydın, Tural Muradlı, Barış Timur, Emre Yaşar, Mete Gürsoy, Ünal Aydın

**Affiliations:** Department of Cardiovascular Surgery, Istanbul Mehmet Akif Ersoy Thoracic and Cardiovascular Surgery Hospital, University of Health Sciences, Istanbul, Turkey; Department of Cardiovascular Surgery, Elazig Fethi Sekin Suam, University of Health Sciences, Elazig, Turkey; Department of Cardiovascular Surgery, Istanbul Mehmet Akif Ersoy Thoracic and Cardiovascular Surgery Hospital, University of Health Sciences, Istanbul, Turkey; Department of Cardiovascular Surgery, Istanbul Mehmet Akif Ersoy Thoracic and Cardiovascular Surgery Hospital, University of Health Sciences, Istanbul, Turkey; Department of Cardiovascular Surgery, Istanbul Mehmet Akif Ersoy Thoracic and Cardiovascular Surgery Hospital, University of Health Sciences, Istanbul, Turkey; Department of Cardiovascular Surgery, Istanbul Mehmet Akif Ersoy Thoracic and Cardiovascular Surgery Hospital, University of Health Sciences, Istanbul, Turkey; Department of Cardiovascular Surgery, Istanbul Mehmet Akif Ersoy Thoracic and Cardiovascular Surgery Hospital, University of Health Sciences, Istanbul, Turkey; Department of Cardiovascular Surgery, Istanbul Mehmet Akif Ersoy Thoracic and Cardiovascular Surgery Hospital, University of Health Sciences, Istanbul, Turkey; Department of Cardiovascular Surgery, Istanbul Mehmet Akif Ersoy Thoracic and Cardiovascular Surgery Hospital, University of Health Sciences, Istanbul, Turkey

**Keywords:** robotic surgery, mitral valve replacement, learning curve, CUSUM analysis

## Abstract

**Objectıves:**

Robot-assisted mitral valve replacement has been shown to be comparable to conventional surgery in terms of safety and efficacy. Our institution has performed robot-assisted mitral valve replacement using the Da Vinci Surgical System for over a decade. This study aimed to evaluate the time-related evolution of clinical outcomes and the impact of the surgical learning curve.

**Methods:**

Patients who underwent robot-assisted mitral valve replacement between July 2013 and January 2024 were evaluated. All procedures were performed by 4 surgeons certified in robotic cardiac surgery, each with prior experience of more than 100 conventional mitral valve replacements. To assess the learning curve, cumulative sum analysis was conducted on cardiopulmonary bypass time and the Mitral Surgery Complexity Score.

**Results:**

A total of 233 patients were included in the analysis. The mean patient age was 48.4 (13.9) years; 117 (50.2%) were male. The mean cardiopulmonary bypass time was 170.3 (55.1) min. Cumulative sum analysis of cardiopulmonary bypass time revealed 3 phases: a learning phase (cases 1-27), a proficiency phase (cases 28-92), and a mastery phase (cases 93 onward). Mitral Surgery Complexity Scores decreased during the early phase, followed by an increase after case 92, indicating a transition towards more complex cases.

**Conclusions:**

Robot-assisted mitral valve replacement has a measurable learning curve, with surgical efficiency and case complexity evolving over time. Approximately 93 procedures appear necessary to achieve operative stability and to confidently expand indications to include more complex patients.

## Graphical abstract

**Figure:**
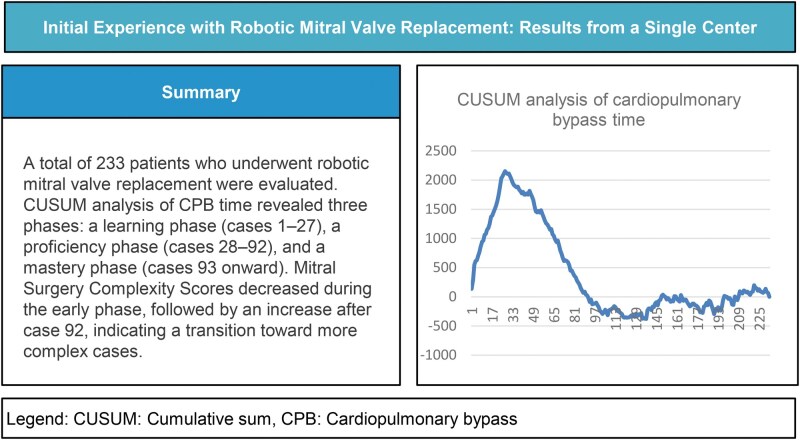

## INTRODUCTION

Prosthetic valve replacement may improve survival in patients with mitral valve pathology for whom mitral valve repair is not feasible or appropriate.^1^ The robot-assisted system has been successfully performed in mitral valve surgery for 25 years.^2^ Robot-assisted mitral valve replacement is not inferior to conventional surgery in terms of effectiveness and safety. It is also superior to conventional surgery in terms of surgical exposure and postoperative comfort.^3–5^

Our institution has been performing robotic mitral valve replacement (MVR) via the Da Vinci Robotic Surgery system for over a decade, offering a unique opportunity to examine temporal trends in surgical performance in a high-volume centre. In this study, we retrospectively analysed over 200 patients who underwent robotic MVR between 2013 and 2024. Our aim was to investigate the time-related evolution in clinical results and to highlight the effects of the surgical learning curve and system advances over the years.

## PATIENTS AND METHODS

### Study design

This study was conducted in accordance with the Declaration of Helsinki and was approved by the hospital’s local ethics committee (approval no. 20250660). Because of the retrospective nature of the study, the need for individual patient consent was waived. No biobanks or data repositories were established for indefinite use in this study. In this single-centre observational study, we evaluated patients who underwent robotic cardiac surgery at our institution between July 2013 and January 2024. There were no formal exclusion criteria for the study; however, patients with moderate to severe aortic insufficiency, significant aorto-iliac disease, femoral artery diameter less than 7 mm, or those requiring redo cardiac surgery were deemed unsuitable for robotic intervention. During this period, a total of 952 patients were operated on using the Da Vinci robotic surgical system. Of these, 341 patients were diagnosed with mitral valve pathology requiring surgical intervention. Among this subgroup, 233 patients underwent robotic mitral valve replacement.

### Preoperative, operative, and postoperative data

Preoperative demographic characteristics and comorbidities of the patients were collected from the hospital’s electronic medical record system. Detailed preoperative echocardiographic parameters—including left ventricular function, valvular morphology, and chamber dimensions—were systematically reviewed.

Intraoperative data were also analysed, including the type and frequency of concomitant procedures performed during surgery, aortic cross-clamp times, and cardiopulmonary bypass (CPB) time. The types of cardioplegia solutions used were also recorded.

Postoperative complications were classified according to established clinical criteria. Renal outcomes included acute kidney injury, defined as an increase of >50% in serum creatinine level from the preoperative value, and requirement for renal replacement therapy (dialysis). Neurological events were categorized as transient or permanent cerebrovascular events. Pulmonary complications such as pleural effusion, pneumothorax, and diaphragmatic paralysis were documented based on radiographic and clinical findings. Infectious complications included postoperative pneumonia, groin wound infections, and prosthetic valve endocarditis. Cardiac-related postoperative events included myocardial infarction, atrial fibrillation, pericardial effusion, and the need for temporary or permanent pacemaker implantation. Additional surgical outcomes assessed were reoperation due to bleeding. In-hospital and late mortality, as well as readmission rates, were recorded. Quantitative postoperative parameters such as total drainage volume (mL), length of hospital stay (days), blood product usage (units), ventilation time (hours), and intensive care unit (ICU) stay (hours) were also extracted. Follow-up transthoracic echocardiography was used to assess postoperative cardiac function.

### Surgical strategies

All operations were performed by 4 console and port surgeons, each certified in robotic cardiac surgery and with prior experience of over 100 conventional mitral valve replacements. The surgeons’ periods of active participation in the robotic program varied over the study duration: Surgeon 1 performed 35 cases between 2013 and 2016, Surgeon 2 performed 70 cases between 2016 and 2022, Surgeon 3 performed 71 cases between 2017 and 2024, and Surgeon 4 performed 57 cases between 2022 and 2024. Cases were performed as they became available based on patient scheduling and clinical suitability. Despite variations in annual volume per surgeon, the cumulative experience and consistent team coordination allowed for observable learning curve dynamics, as reflected in the CUSUM analysis.

All procedures were performed under general anaesthesia. A single-lumen endotracheal tube was used for airway management, and a transoesophageal echocardiography probe was inserted. CPB access was obtained via direct surgical cannulation of the femoral artery and vein, and percutaneous cannulation of the right internal jugular vein.^6^ CPB was initiated following systemic heparinization. A limited pericardiotomy was carried out under robotic visualization, taking care to preserve the integrity of the phrenic nerve. The ascending aorta was visualized and vented through the aortic root. A transthoracic aortic cross-clamp (Chitwood clamp; Aesculap Inc., Center Valley, PA, USA) was applied via second intercostal space, and cardiac arrest was achieved using antegrade cold cardioplegia. The interatrial (Sondergaard) groove was dissected to expose the left atrium. Left atriotomy was performed. A robotic atrial retractor was inserted to ensure optimal exposure of the mitral valve apparatus. The subvalvular apparatus was preserved whenever technically feasible, in order to maintain left ventricular geometry and optimize postoperative ventricular function. After excising the diseased native mitral valve, the prosthetic valve was implanted using everting pledgeted sutures in an anti-anatomical position under direct robotic visualization. Once implantation was completed, the left atrium was closed in standard fashion. Following rewarming and gradual weaning from CPB, cardiac and prosthetic valve function were reassessed using TEE. Once satisfactory haemodynamic parameters were achieved, CPB was discontinued. The robotic instruments were withdrawn, and chest drains were placed in the pericardial and right pleural spaces. All port sites were closed in layers.

### Mitral surgery complexity score

Raposeiras-Roubin et al.^7^ developed the MitraScore as a user-friendly clinical tool to predict mortality risk in patients undergoing transcatheter edge-to-edge mitral valve repair. This scoring system is composed of 8 clinical variables, each assigned 1 point, and has demonstrated good discriminatory power in risk stratification.

Inspired by the structure and simplicity of the MitraScore, we aimed to develop a comparable risk assessment model specifically for patients undergoing robotic MVR. The Mitral Surgery Complexity Score (MSCS) is composed of 7 preoperative clinical variables, each assigned 1 point. This cumulative score is intended to reflect the overall surgical risk profile of the patient. The components of MSCS are detailed in Table 1.

**Table 1.** The Components of the Mitral Surgery Complexity Score

Variable	Score
Concomitant cardiac surgical procedure	1
New York Heart Association functional classification III-IV	1
Chronic obstructive pulmonary disease	1
Chronic kidney disease (elevated creatinine)	1
Left ventricular ejection fraction <45%	1
Pulmonary artery pressure >40 mmHg	1
Mitral annular calcification	1
**Total score (0-7)**	

Higher total scores are associated with increased operative complexity and a greater likelihood of postoperative complications or adverse outcomes. As such, this score serves not only as a surgical risk stratification tool but also as an indicator of how surgical case selection has evolved over time.

The MSCS was designed as a simple, institution-specific tool to reflect operative complexity. It has not yet been formally validated and is used here for descriptive purposes.

### Statistical analysis

CUSUM analysis was performed to evaluate the trends in CPB time and MSCS as cases progressed chronologically. CPB time was defined as the duration from the initiation to the termination of cardiopulmonary bypass. The total MSCS was calculated by assigning one point for each of the predefined risk factors. For both parameters, CUSUM was calculated as the cumulative sum of the differences between each individual case value and the overall mean value. The CUSUM for the first case was calculated as the parameter value minus the overall mean. For each subsequent case, CUSUM was determined using the following formulas: CUSUM_*n*_ = CUSUM_*n*__−1_ + (CPB time_*n*_ − mean CPB time) for CPB time, and CUSUM_*n*_ = CUSUM_*n*__-1_ + (MSCS_*n*_ − mean MSCS) for the MSCS. CUSUM charts were plotted using line graphs generated in Microsoft Excel 2024 (Microsoft Corporation, Redmond, WA) to visualize learning curves and temporal trends.

Statistical analyses were performed on R version 4.0.3 (R Foundation for Statistical Computing). Normally distributed continuous data were presented as mean and standard deviation (SD). Non-normally distributed continuous data were presented as median, first, and third interquartile range (Q1-Q3). Categorical data were presented as the number of patients with a ratio. Categorical variables were compared among the 4 groups using the Chi-square test. Continuous variables were tested for normality with the Shapiro-Wilk test. Normally distributed variables were analysed with one-way ANOVA, while non-normally distributed variables were analysed with the Kruskal-Wallis test. A *P* value <.05 was considered statistically significant.

## RESULTS

The mean age was 48.4 (13.9) years, and 117 patients (50.2%) were male. The most common comorbidities included diabetes mellitus (14.6%), hypertension (17.7%), and chronic obstructive pulmonary disease (13.7%). None of the patients were on dialysis, although 3.9% had elevated creatinine levels consistent with chronic kidney disease. Patient demographics and comorbidities are detailed in Table 2.

**Table 2.** Patient Demographics and Comorbidities

Variable	All patients, *n*: 233	Learning phase, *n*: 27	Proficiency phase, *n*: 65	Mastery phase, *n*: 141	*P* value
Age (years)	48.4 (13.9)	42.6 (15.2)	45.4 (13.5)	51.0 (13.5)	.01
Sex (male)	117 (50.2%)	15 (55.6%)	29 (44.6%)	73 (51.8%)	.53
Body surface area (m²)	1.82 (0.19)	1.79 (0.17)	1.82 (0.21)	1.83 (0.19)	.63
Cerebrovascular event	1 (0.4%)	0 (0.0%)	0 (0.0%)	1 (0.7%)	.72
Diabetes mellitus	34 (14.6%)	4 (14.8%)	6 (9.2%)	24 (17.0%)	.34
Hypertension	41 (17.7%)	6 (23.1%)	11 (16.9%)	24 (17.0%)	.75
Coronary stent	2 (0.9%)	1 (3.7%)	0 (0.0%)	1 (0.7%)	.21
Chronic kidney disease (elevated creatinine)	9 (3.9%)	0 (0.0%)	1 (1.5%)	8 (5.7%)	.19
Chronic obstructive pulmonary disease	32 (13.7%)	0 (0.0%)	4 (6.2%)	28 (19.9%)	.01
Permanent atrial fibrillation	26 (11.2%)	3 (11.1%)	5 (7.7%)	18 (12.8%)	.56
Paroxysmal atrial fibrillation	6 (2.6%)	0 (0.0%)	0 (0.0%)	6 (4.3%)	.13

The mean preoperative ejection fraction (EF) of the cohort was 56.4% (8.2), and the mean pulmonary artery pressure was 35.9 (12.5) mmHg. Moderate-to-severe tricuspid regurgitation (grades 3 and 4) was observed in 4.7% of patients. Rheumatic disease accounted for the majority of mitral valve pathologies (63.9%), followed by degenerative causes (35.6%), including Barlow-type mitral valve disease (11.2%). Detailed preoperative echocardiographic and clinical findings are presented in Table 3.

**Table 3.** Preoperative Echocardiographic and Clinical Findings

Variable	All patients, *n*: 233	Learning phase, *n*: 27	Proficiency phase, *n*: 65	Mastery phase, *n*: 141	*P* value
Ejection fraction (%)	56.4 (8.2)	59.2 (5.2)	59.3 (6.1)	54.5 (9.0)	.01
Pulmonary artery pressure (mmHg)	35.9 (12.5)	31.4 (8.0)	33.3 (10.2)	38.1 (13.8)	.01
Tricuspid regurgitation—Grade 3	10 (4.3%)	1 (3.7%)	0 (0.0%)	9 (6.4%)	.14
Tricuspid regurgitation—Grade 4	1 (0.4%)	0 (0.0%)	0 (0.0%)	1 (0.7%)	
Rheumatic mitral valve disease	149 (63.9%)	15 (55.6%)	44 (67.7%)	90 (63.8%)	.54
Degenerative mitral valve disease	83 (35.6%)	12 (44.4%)	21 (32.3%)	50 (35.5%)	.54
Barlow’s mitral valve disease	26 (11.2%)	1 (3.7%)	2 (3.1%)	23 (16.3%)	.01
Mitral valve infective endocarditis	2 (0.9%)	1 (3.7%)	1 (1.5%)	0 (0.0%)	.13
Mitral annular calcification	8 (3.4%)	0 (0.0%)	0 (0.0%)	8 (5.7%)	.07
Left atrium diameter (mm)	49.2 (9.9)	51.0 (8.8)	45.2 (7.0)	51.0 (11.0)	.01
Left ventricular end-diastolic diameter (mm)	52.5 (7.1)	56.5 (7.2)	51.52 (8.1)	52.2 (6.3)	.01

The mean cardiopulmonary bypass time was 170.3 (55.1) min, and the aortic cross-clamp time was 110.5 (39.5) min. Mechanical prosthesis were implanted in 84.5% of patients, and biological prosthesis in 15.5%. Concomitant procedures included tricuspid valve repair (10.3%), radiofrequency ablation (7.3%), left atrial appendage closure (6.4%), and mass excision (6.0%). Custodiol cardioplegia was used in 74.7% of cases, and Del Nido in 25.3%. Intraoperative data are presented in Table 4.

**Table 4.** Intraoperative Characteristics

Variable	All patients, *n*: 233	Learning phase, *n*: 27	Proficiency phase, *n*: 65	Mastery phase, *n*: 141	*P* value
Aortic cross-clamp time (min)	110.5 (39.5)	172.6 (44.5)	89.7 (22.6)	108.1 (32.2)	.01
Cardiopulmonary bypass time (min)	170.3 (55.1)	250.2 (54.6)	137.0 (34.1)	170.4 (46.9)	.01
Ablation performed	17 (7.3%)	8 (29.6%)	8 (12.3%)	1 (0.7%)	.01
Atrial septal defect repair	8 (3.4%)	0 (0.0%)	0 (0.0%)	8 (5.7%)	.07
Mass excision	14 (6.0%)	1 (3.7%)	7 (10.8%)	6 (4.3%)	.16
Left atrial appendage closure	15 (6.4%)	8 (29.6%)	7 (10.8%)	0 (0.0%)	.01
Tricuspid valve repair	24 (10.3%)	4 (14.8%)	2 (3.1%)	18 (12.8%)	0.07

The most common postoperative complication was atrial fibrillation, occurring in 10.7% of patients. Pleural effusion was observed in 6.9%, while 3.0% required reoperation due to bleeding. Acute kidney injury was identified in 2.1% of patients, with only one requiring dialysis. Neurological events were rare, with 0.9% experiencing transient cerebrovascular events and no cases of permanent cerebrovascular injury. In-hospital mortality was 2.6%. The in-hospital mortality rate was 2.6%, with the 6 cases of mortality occurring in the 5th, 20th, 79th, 95th, 171st, and 210th cases, respectively. Among the 6 in-hospital mortalities, the causes included bleeding (*n*: 1), myocardial infarction (*n*: 1), cardiac tamponade (*n*: 1), and multi-organ failure (*n*: 3). One patient (81st) experienced an iliac artery injury during decannulation, which required surgical repair. Minor groin hematomas occurred in 3 patients and resolved without intervention. No other major vascular complications, such as limb ischaemia or vessel perforation, were observed. Postoperative outcomes and complications are detailed in Table 5.

**Table 5.** Postoperative Outcomes and Complications

Variable	All patients, *n*: 233	Learning phase, *n*: 27	Proficiency phase, *n*: 65	Mastery phase, *n*: 141	*P* value
New onset atrial fibrillation	25 (10.7%)	2 (7.4%)	1 (1.5%)	22 (15.6%)	.01
Pericardial effusion	7 (3.0%)	1 (3.7%)	4 (6.2%)	2 (1.4%)	.18
Pleural effusion	16 (6.9%)	1 (3.7%)	1 (1.5%)	14 (9.9%)	.07
Pneumothorax	5 (2.1%)	0 (0.0%)	2 (3.1%)	3 (2.1%)	.65
Acute kidney injury—dialysis	1 (0.4%)	0 (0.0%)	1 (1.5%)	0 (0.0%)	.27
Acute kidney injury—elevated creatinine	5 (2.1%)	0 (0.0%)	1 (1.5%)	4 (2.8%)	.60
Transient cerebrovascular events	2 (0.9%)	0 (0.0%)	1 (1.5%)	1 (0.7%)	.73
Permanent cerebrovascular events	0 (0%)	0 (0.0%)	0 (0.0%)	0 (0.0%)	NA
Diaphragmatic paralysis	3 (1.3%)	0 (0.0%)	0 (0.0%)	3 (2.1%)	.37
Endocarditis	3 (1.3%)	1 (3.7%)	1 (1.5%)	1 (0.7%)	.44
Wound infection	3 (1.3%)	1 (3.7%)	0 (0.0%)	2 (1.4%)	.35
Pneumonia	10 (4.3%)	1 (3.7%)	0 (0.0%)	9 (6.4%)	.11
Reoperation due to bleeding	7 (3.0%)	1 (3.7%)	0 (0.0%)	6 (4.3%)	.25
Myocardial infarction	1 (0.4%)	0 (0.0%)	1 (1.5%)	0 (0.0%)	.27
Permanent pacemaker	4 (1.7%)	1 (3.7%)	2 (3.1%)	1 (0.7%)	.33
Temporary pacemaker	12 (5.2%)	2 (7.4%)	3 (4.6%)	7 (5.0%)	.85
Drainage volume (mL)	319.6 (145.1)	321.7 (122.3)	295.3 (104.2)	337.5 (173.5)	.22
Blood product usage (units)	0.0 [0.0, 1.0]	1.0 [0.0, 2.0]	0.5 [0.0, 1.0]	0.0 [0.0, 1.0]	.04
Ventilator time (hours)	11.0 [5.0, 17.0]	15.0 [10.0, 20.0]	11.0 [6.0, 16.0]	10.0 [5.0, 14.0]	.01
Intensive care unit stay (hours)	18.0 [24.0, 48.0]	36.0 [24.0, 60.0]	24.0 [18.0, 48.0]	18.0 [12.0, 36.0]	.01
Length of hospital stay (days)	6.0 [5.0, 9.0]	8.0 [6.0, 10.0]	6.0 [5.0, 8.0]	6.0 [4.0, 7.0]	.03
In-hospital mortality	6 (2.6%)	2 (7.4%)	1 (1.5%)	3 (2.1%)	.23
Readmission	3 (1.3%)	0 (0.0%)	0 (0.0%)	3 (2.1%)	.37

**Figure 1.**
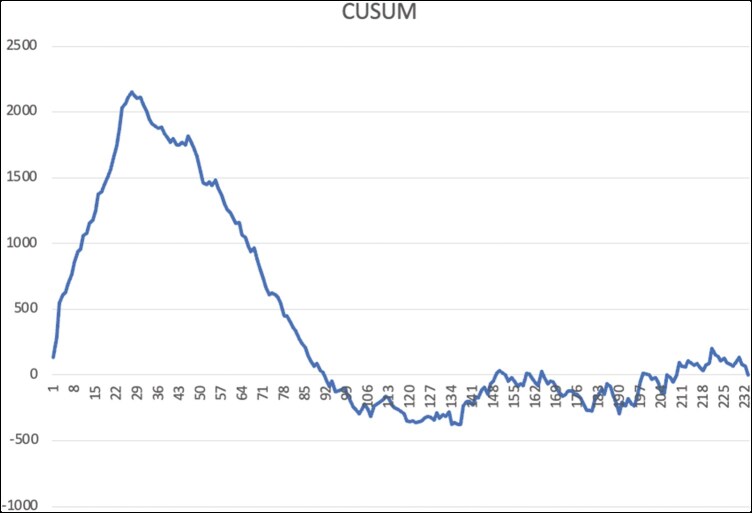
CUSUM Analysis of Cardiopulmonary Bypass (CPB) Time.

**Figure 2.**
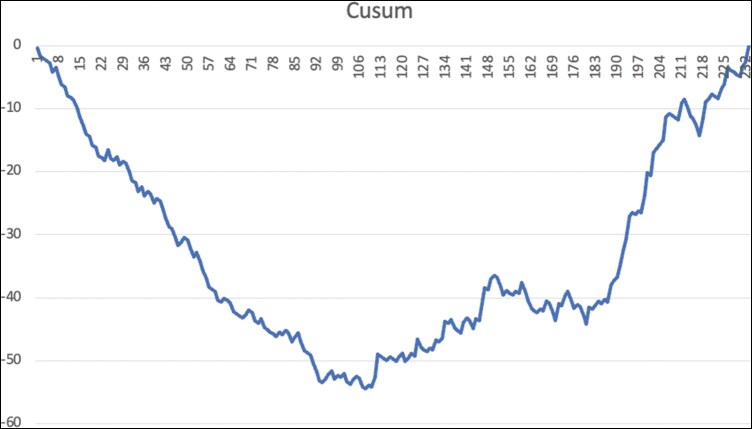
CUSUM Analysis of Mitral Surgery Complexity Score.

Figure 1 presents the CUSUM analysis of CPB time plotted against case number. The curve demonstrates an initial increase up to case 27, followed by a decreasing trend between cases 27 and 92, and subsequently plateaus after case 92. The negative slope observed during this phase indicates a reduction in CPB time as the surgical experience increased, with the plateau suggesting stabilization of operative times beyond case 92. The mean MSCS was 1.36 ± 1.22, median MSCS was 1. The interquartile range 1 was 0, to the interquartile range 3 was 2. Figure 2 depicts the CUSUM analysis of the Mitral Surgery Complexity Score according to case number. A negative slope is observed in the first 92 cases, which reverses to a positive slope after case 92. This shift indicates that as experience accumulates, increasingly complex cases are selected for robotic mitral valve surgery.

## DISCUSSION

The acquisition of new surgical techniques, particularly those involving advanced technologies such as robotic platforms, typically entails a learning curve. This curve reflects a progressive increase in the surgeon’s familiarity with the technical nuances and intraoperative challenges of the robotic approach. As experience accumulates, operative times generally decrease, complication rates tend to diminish, and case selection evolves to include more complex pathologies.^8^

Although most learning curve studies in robotic cardiac surgery focus on mitral valve repair, there is limited data on robotic mitral valve replacement.^9–12^ In the present study, we evaluated the learning curve for robotic MVR using CUSUM analysis. The CUSUM plot of CPM time delineated 3 distinct phases: an initial learning phase (cases 1-27), a proficiency phase (cases 28-92), and a mastery phase (cases 93 and onward). These inflection points reflect not only a temporal improvement in surgical efficiency but also a shift in the complexity of cases selected for robotic intervention, as evidenced by the increasing Mitral Surgery Complexity Score after the 93rd case. Our findings suggest that approximately 93 procedures are required to attain operative proficiency comparable to conventional MVR.

The proficiency phase (cases 28-92) was marked by a steep reduction in operative time, likely attributable to increased familiarity with the robotic platform and standardization of the surgical workflow. During this period, simpler pathologies and lower-risk patients were preferentially selected, in line with recommendations for initiating robotic programs with well-defined, low-complexity cases. In particular, cases with extensive annular calcification were avoided due to technical limitations of the robotic platform. The strength of robotic instruments may preclude safe annular decalcification, and the lack of tactile feedback makes it difficult to assess the extension of calcification into the ventricle.^13^

Our results align with previous reports that have demonstrated robotic mitral surgery to be a safe and effective alternative to conventional approaches, offering favourable short-term outcomes.^14^ Long-term outcomes from other surgical teams have also confirmed the feasibility and safety of robotic MVR.^12–15^ A major reason for the predominance of valve replacement in our robotic mitral program is the high prevalence of rheumatic mitral valve disease in our patient population. Additionally, mitral valve replacement was preferred in degenerative cases where repair was not feasible. Similarly, a report from the United States showed that between 2012 and 2019, among 53 117 interventions performed for degenerative mitral regurgitation, 16 500 were mitral valve replacements.^16^

The literature suggests a variable number of cases required to reach the plateau phase in robotic cardiac procedures. For example, Güllü et al.^9^ reported that the learning curve for robotic mitral valve repair matured after approximately 30 cases. On the other hand, Goodman et al.^17^ reported significant improvements in CPB, cross-clamp, and total operative times after the initial 200 cases.

Robotic MVR generally offers advantages in terms of enhanced visualization and precision, potentially improving exposure of the mitral apparatus and facilitating more delicate surgical manoeuvres. Operative times for robotic MVR are initially longer than conventional sternotomy due to the learning curve and setup requirements, but they tend to decrease with experience, as demonstrated in our CUSUM analysis. Minimally invasive non-robotic approaches, such as right mini thoracotomy, offer reduced surgical trauma compared to sternotomy, yet they may be limited by visualization and instrument dexterity. Learning curve dynamics have also been reported for other minimally invasive mitral approaches. For instance, Wu et al.^18^ analysed the first 100 patients who underwent mitral valve replacement via a right anterolateral minithoracotomy and found that at least 33 cases were required for the surgeon to reach a performance plateau. Further, Gillinov et al.^19^ observed progressive reductions not only in operative times but also in stroke incidence, ICU stay, and total hospital length of stay. During our study period, the COR-KNOT device was not utilized for prosthetic valve fixation. However, a recent study demonstrated that its use in the final 10 cases reduced knotting time from 30 min to 6 min, suggesting a potential for significantly shorter CPB time. Incorporating the COR-KNOT system may therefore contribute to improved operative efficiency and potentially accelerate the learning curve in robotic mitral valve surgery.^12^

The observed increase in the complexity of cases beyond the 93rd procedure in our series further supports the notion that surgical confidence and competence facilitate the expansion of indications for robotic MVR. During the mastery phase, the Mitral Surgery Complexity Score showed a positive slope, indicating a deliberate shift towards operating on higher-risk patients with more complex pathologies. This suggests that growing surgical confidence contributed to a greater willingness to perform robotic procedures even in higher-risk cases.

Operative efficiency in robotic surgery has been shown to depend not only on the experience of the primary surgeon but also on the collective coordination of the entire surgical team.^20–22^ In our experience, the robotic cardiac surgery team consisted of 4 surgeons, all certified in robotic cardiac procedures. Throughout the study period, we maintained a stable group of perfusionists and scrub nurses who were familiar with the specific requirements of robotic cases. Newly assigned operating room nurses, even those experienced in open-heart surgery, underwent additional training before being integrated into the robotic team. This structured team approach facilitated smoother case execution and likely contributed to the progressive improvements observed across the learning curve. In addition, wet-lab simulation training has been shown to facilitate the acquisition of key robotic skills, particularly for surgeons and team members without prior robotic experience.^23^

This study has several limitations. First, it is retrospective in nature. Second, all procedures were performed at a single centre but by multiple surgeons, and thus the findings reflect the collective experience of the institution rather than a single operator. Additionally, the Mitral Surgery Complexity Score used in this analysis has not been previously validated and was developed based on institutional consensus and expert opinion. It should therefore be interpreted as a descriptive measure of temporal case complexity rather than a fully validated prognostic model. Future work will focus on internal and external validation in independent cohorts. Furthermore, during the study period, the surgical team performed various robotic cardiac procedures beyond mitral valve replacement, which may have contributed to a broader refinement of robotic skills and team coordination.

## CONCLUSION

This study demonstrates that robotic mitral valve replacement can be safely and effectively implemented with a structured, stepwise learning process. Through CUSUM analysis, we identified 3 distinct phases—learning, proficiency, and mastery—corresponding to improved surgical efficiency and increasing case complexity. Our findings suggest that approximately 93 cases are required to reach operative stability and enable the confident inclusion of more complex patients. The successful maturation of a robotic mitral program depends not only on the individual surgeon’s adaptation but also on cohesive team dynamics, strategic case selection, and institutional commitment. These insights may guide other centres aiming to establish or advance their own robotic cardiac surgery initiatives. Overall, this study contributes valuable insight into the learning dynamics of robotic MVR and may serve as a reference for other centres aiming to implement or optimize similar programs.

## Data Availability

The data that support the findings of this study are available from the corresponding author upon reasonable request.
